# Dynamic changes of neutrophil-to-lymphocyte ratio and platelet-to-lymphocyte ratio predicts breast cancer prognosis

**DOI:** 10.1186/s12885-020-07700-9

**Published:** 2020-12-07

**Authors:** Ju-Yeon Kim, Eun Jung Jung, Jae-Myung Kim, Han Shin Lee, Seung-Jin Kwag, Ji-Ho Park, Taejin Park, Sang-Ho Jeong, Chi-Young Jeong, Young-Tae Ju

**Affiliations:** 1 Department of Surgery, Gyeongsang National University School of Medicine and Gyeongsang National University Hospital, 11, Samjeongjaro, Seongsangu, Changwonsi, Republic of Korea 51472; 2 Department of Surgery, Gyeongsang National University School of Medicine and Gyeongsang National University Changwon Hospital, 11, Samjeongjaro, Seongsangui, Changwonsi, Republic of Korea

**Keywords:** Neutrophil-to-lymphocyte ratio, Platelet-to-lymphocyte ratio, Prognostic factor, Biomarker

## Abstract

**Background:**

We aimed to identify whether neutrophil-to-lymphocyte ratio (NLR) and platelet-to-lymphocyte ratio (PLR) are more useful predictors after initial intention to treat than at the time of diagnosis.

**Methods:**

We collected the medical data of 533 patients. The results of the peripheral blood sampling before the primary treatments were labeled as initial cohort, and those obtained between 24 and 36 months after initial treatment were defined as the 2nd cohort. Delayed metastasis has been defined as distant metastasis 2 years after treatment, and survival outcome was estimated and compared across groups.

**Results:**

Median follow-up duration was 74 months (24–162 months), and 53 patients experienced delayed metastasis. In univariate analysis, metastasis-free survival, patient age at diagnosis, tumor size, axillary lymph node metastasis, HER-2 status, initial NLR and PLR, and 2nd NLR and PLR were found to be significantly associated with delayed metastasis. However, in multivariate analysis, only the 2nd NLR and PLR were found to be significantly associated with delayed metastasis, excluding initial NLR and PLR. Metastasis-free survival was analyzed through the pattern changes of NLR or PLR. The results revealed that patients with continued low NLR and PLR values at pre- and post-treatment (low initial values and 2nd values) showed a significantly better prognosis than those with a change in value or continued high NLR and PLR.

**Conclusions:**

We identified that patients with persistent high NLR and PLR after initial treatment have significant worse prognosis in terms of late metastasis. Therefore, these results suggest that NLR and PLR are more useful in predicting prognosis post-treatment.

## Background

Breast cancer is a common malignancy in women around the world, and despite the availability of optimal local and systemic therapies, a substantial number of women with breast cancer will develop systemic recurrence [1]. Indeed, the leading cause of breast cancer-related deaths is its metastatic spread, although the timing and distribution of breast cancer metastases vary considerably.

Previous studies have reported that there is a significant difference in the onset of recurrence depending on the hormone receptor status and hormonal therapy, wherein estrogen receptor-negative tumors are generally associated with early recurrence [2, 3]. The mechanisms that account for the wide variability in the propensity of breast cancer to metastasize are currently unknown. However, metastatic spread form a primary breast tumor can occur at an early, pre-symptomatic stage, and disseminated cells often settle in the bone marrow where they can lie dormant for years before becoming clinically evident [4].

In cancer patients, inflammatory cells and their mediators in the tumor microenvironment are considered to play an important role in cancer development and progression. A recent meta-analysis demonstrated that an elevated peripheral neutrophil-to-lymphocyte ratio (NLR) and platelet-to-lymphocyte ratio (PLR) at the baseline before the first treatment represent poor prognostic factors in breast cancer [5–8]. Inflammatory conditions can be migrated through treatments and lifestyle changes. In particular, chemotherapy affects various cells, including inflammatory and immune cells, and the subsequent recovery process may vary from patient to patient; these post-treatment changes may then affect the expression of disseminated metastatic cells [9].

Most previous studies have conducted primary tumor or blood tests before treatment; meanwhile, studies evaluating test results obtained after treatment as prognostic markers remain to be limited. The status of tumors or patients after treatment can also be useful surrogate markers of prognosis, for example, complete remission after neoadjuvant chemotherapy or Ki-67 level after preoperative endocrine therapy [10–12].

The primary aim of this study is to determine whether the NLR and PLR obtained after initial intention to treat could predict prognosis after 2 years in patients without evidence of early cancer recurrence or metastasis.

## Methods

### Study cohort

The data of female patients with primary breast cancer who diagnosed from January 2006 to December 2015 at a single institute were analyzed for this retrospective cohort study. All patients were recommended to undergo treatment with standard adjuvant therapy and post treatment surveillance according to the guidelines. The exclusion criteria were as follows: patients who were concurrently or previously diagnosed with other organs malignancies; patients who had M1 diseases on diagnosis; patients who had systemic autoimmune disease, such as systemic lupus or scleroderma; and patients who had incomplete data. The characteristics of study patients are presented in Table 1. Out of the 674 patients assessed, 533 patients were included in the final analysis; of these, 29 (4.3%) had disease recurrence or distant metastasis before 2 years, 51 (7.6%) were lost to follow-up within 2 years, and 61 (9.1%) had no blood test results between 24 and 36 months. This study protocol was received approval from the institutional review board and met the guidelines of the responsible governmental agencies (IRB No. GNUH 2020–04-020). Informed consent was waived based on the retrospective format of this study.

**Table 1:** Clinicopathological characteristics of initial cohort patients

		Number	(%)
Age	(mean ± SD, years)	52.10 ± 11.10	
	≤50	336	49.9
	> 50	338	50.1
Stage	I	290	43.1
	II	280	41.5
	III	104	15.4
Estrogen receptor	Negative	231	34.3
	Positive	443	65.7
Progesterone receptor	Negative	288	42.7
	Positive	386	57.3
Operation type	Conservation	395	58.6
	Mastectomy	279	41.4
Chemotherapy	None	105	15.6
	AC	148	22.0
	AC-Taxans	93	13.8
	FAC	178	26.4
	TAC	58	8.6
	Others	92	13.6
Initial hormone therapy	None	195	28.9
	Tamoxifen	306	45.4
	Aromatase inhibitor	173	25.7

*AC* Adriamycin and Cyclophosphamide, *FAC* Fluorouracil, Adriamycin and Cyclophosphamide, *TAC* Taxan, Adriamycin and Cyclophosphamide

### Data collection and statistical analysis

The NLR and PLR are defined as the absolute neutrophil count or absolute platelet count divided by the absolute lymphocyte count. Peripheral blood samplings were performed both at the initial work-up period before treatment and at routine follow-up. The results obtained before the primary treatments were labeled as the initial cohort; meanwhile the results derived between 24 and 36 months were defined as the 2nd cohort. Receiver operating characteristic (ROC) curve analysis was used to determine the cut-off value of the NLR and PLR. Non-parametric tests of the association of cohorts were compared with Mann-Whitney U test.

We reviewed the medical records, pathology reports, and follow-up data of the enrolled patients. Delayed metastasis was defined as metastasis 2 years after initial treatment, and metastasis-free survival (MFS) was defined as the time interval from the date of initial diagnosis to the date of distant metastasis or to the most recent follow-up date with no evidence of distant metastasis. To determine whether NLR and PLR are useful predictors of delayed metastasis, survival outcome was estimated using the Kaplan–Meier curve, and univariate analysis for comparing across groups was performed using the log-rank test. With regard to multivariate analysis, a Cox proportional hazards model was used to estimate the adjusted hazard ratio for significance. All analyses were carried out using SPSS (version 20.0; SPSS, Inc.), and *p* < 0.05 was assumed statistically significant.

## Results

We reviewed the data of 533 patients (stage I; 219(41.1%), stage II; 237 (44.5%) and stage III; 77 (14.4%)) to identify the prognostic factors affecting delayed metastasis. The median follow-up duration was 74 months (range, 24–162 months), and 53 patients were found to experience delayed distant metastasis.

**Fig. 1:**
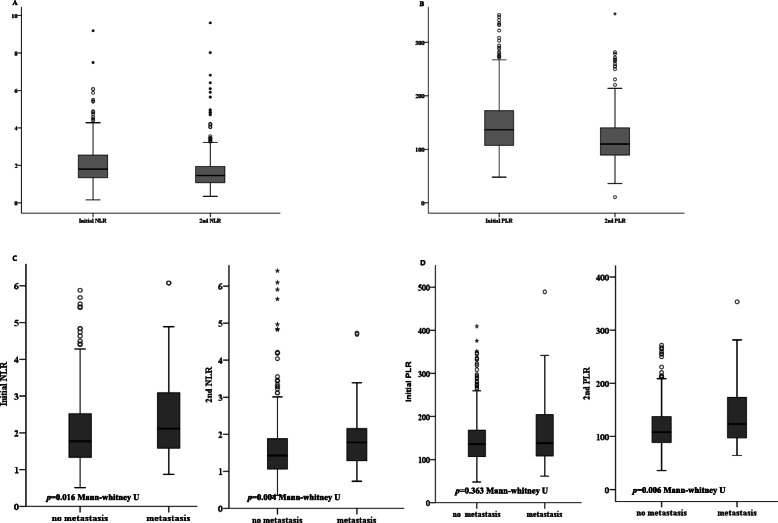
Non-parametric analysis of LNR and PLR. **a** and **b** showed the distribution of initial and 2nd parameters. **c** and **d** showed the average difference in values according to delayed metastasis

The mean values of the initial NLR and PLR were 2.11 and 145.75, respectively, and the mean values of the 2nd NLR and PLR were 1.72 and 118.55, respectively. The NLR and PLR values had a tendency to be lower in the 2nd group than in the initial group (Fig. 1a and b).

The average difference in value was analyzed according to delayed metastasis. Initial NLR and 2ndNLR were significantly higher in the metastasis group than in the no metastasis group. (initial NLR; no metastasis vs. metastasis [mean ± SD = 2.06 ± 1.17 vs. 2.40 ± 1.18] *p* = 0.016; and 2nd LNR; no metastasis vs. metastasis [mean ± SD = 1.65 ± 1.29 vs. 2.36 ± 3.12] *p* = 0.004). The continuous PLR value was also higher in the metastasis group than in the no metastasis group, bot statistically only 2nd PLR value as noted (initial PLR; no metastasis vs. metastasis [mean ± SD = 144.36 ± 54.40 vs. 157.34 ± 73.10] *p* = 0.363; and 2nd PLR; no metastasis vs. metastasis [mean ± SD = 116.22 ± 38.32 vs. 139.66 ± 56.73] *p* = 0.003) (Fig. 1c and d).

ROC curve analysis was also used to determine the optimal cutoff value of NLR and PLR for the initial and 2nd groups. The initial LNR cutoff value was 1.82 (area under the ROC curve [AUC], 0.601; 95% confidence interval [CI], 0.520–0.681) with 64.2% sensitivity and 52.1% specificity. The 2nd LNR cutoff value was 1.76 (AUC, 0.619; 95% CI, 0.540–0.711) with 50.9% sensitivity and 70.6% specificity. The initial PLR cut-off value was 204.27 (AUC, 0.534; 95% CI, 0.494–0.634) with 28.3% sensitivity and 87.7% specificity. The 2nd PNR cut-off value was 112.67 (AUC, 0.625; 95% CI, 0.540–0.711) with 69.8% sensitivity and 55.6% specificity.

The survival outcome was estimated using the Kaplan–Meier method, and the results were compared across groups using the log-rank test (Table 2). Patient age at diagnosis, tumor size, lymph node metastasis, HER-2 status, initial NLR and PLR, and 2nd NLR and PLR were found to be significantly associated with delayed metastasis.

**Table 2:** Univariate analysis with Kaplan–Meier and Cox proportional hazards model of the clinical characteristics affecting delayed metastasis in 2nd cohort

	Number	MFS (mean ± SD, months)	Log-Rank	Univariate HR (±95% CI)
Age (years)			0.034	
≤50	270	149.53 ± 2.57		
> 50	263	135.87 ± 3.18		1.796 (1.036–3.113)
Tumor size (cm)			0.043	
≤2	296	148.20 ± 2.63		
> 2	237	142.36 ± 3.40		1.737 (1.009–2.992)
Lymph node metastasis			0.002	
No	331	149.38 ± 2.46		
Yes	202	139.48 ± 3.78		2.334 (1.351–4.031)
Histologic grade			0.395	
1 and 2	354	141.77 ± 2.47		
3	179	144.54 ± 3.58		
Estrogen receptor			0.088	
Negative	165	151.02 ± 3.52		
Positive	368	143.35 ± 2.58		
Progesterone receptor			0.094	
Negative	220	149.99 ± 3.15		
Positive	313	138.71 ± 2.67		
HER-2			0.005	
Negative	417	148.04 ± 2.37		
Positive	103	118.6 ± 4.10		2.208 (1.247–3.911)
Pre NLR (cut-off 1.82)			0.020	
Low	269	150.01 ± 2.55		
High	264	141.11 ± 3.37		1.920 (1.095–3.367)
Pre PLR (cut-off 204.27)			0.007	
Low	459	148.10 ± 2.10		
High	74	133.19 ± 6.301		2.238 (1.230–4.073)
2nd NLR (cut-off 1.76)			0.001	
Low	365	149.22 ± 2.32		
High	168	137.48 ± 4.31		2.434 (1.420–4.172)
2nd PLR (cut-off 112.67)			< 0.001	
Low	283	151.85 ± 2.31		
High	250	138.70 ± 3.58		2.759 (1.535–4.960)

Most of the patients included in the analysis were not subjected to HER-2 targeted therapy due to medical insurance problems in Korea at the time. Therefore, we performed two multivariate analyses, which included and excluded the HER-2 status (Table 3). The 2nd NLR and PLR were significantly associated with delayed metastasis; however, the initial NLR and PLR showed no prognostic significance.

**Table 3:** Multivariate analysis for delayed MFS

	Her-2 Including analysis		Her-2 excluding analysis	
	Multivariate HR (±95% CI)	*P* value	Multivariate HR (±95% CI)	*P* value
Age	2.091 (1.182–3.699)	0.011	1.877 (1.070–3.295)	0.028
Size		0.418		0.228
LN metastasis	2.046 (1.175–3.564)	0.011	1.985 (1.138–3.464)	0.016
Pre NLR Value		0.144	1.657 (0.911–3.011)	0.098
Pre PLR Value		0.124		0.300
2nd NLR Value	2.231 (1.166–4.268)	0.015	1.897 (1.011–3.557)	0.046
2^nd^ PLR Value	2.371 (1.295–4.341)	0.005	1.968 (1.019–3.800)	0.044
HER-2	2.183 (1.227–3.885)	0.008		

**Fig. 2:**
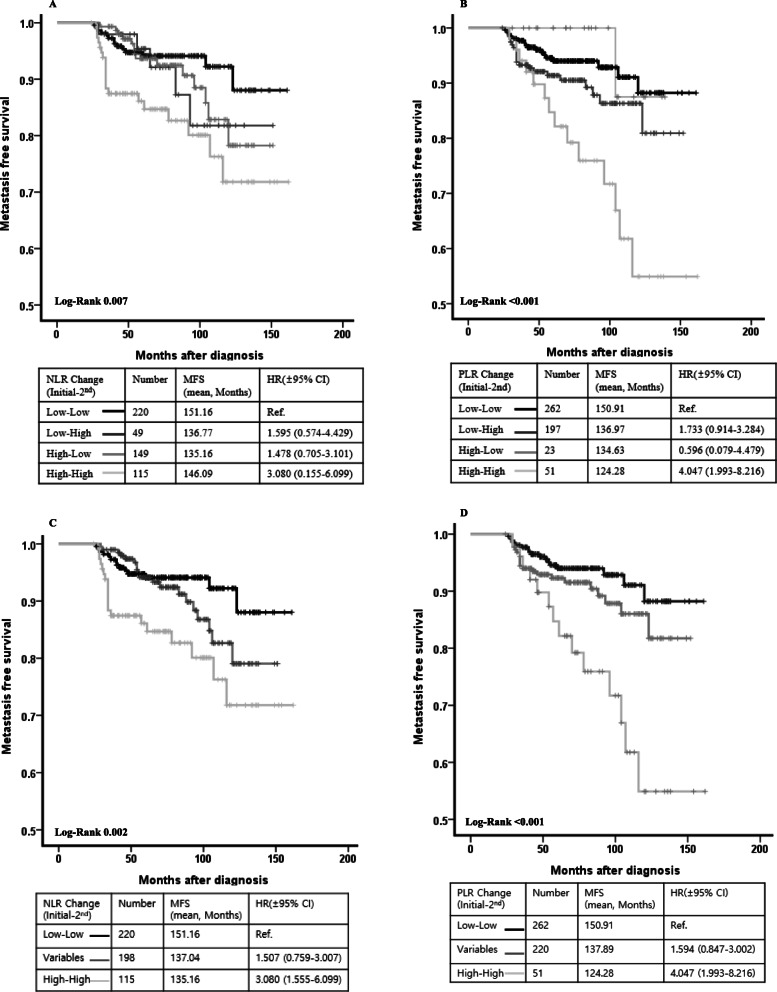
Kaplan-Meier curves and log-rank tests comparing delayed metastasis free survival with different subgroups according to NLR and PLR changes

We examined the effects of NLR and PLR changes after initial treatments, wherein the MFS was analyzed according to the change in NLR or PLR (Fig. 2a and b). Kaplan–Meier analysis revealed that patients with continued low NLR and PLR at the time of diagnosis and after treatment (low initial values and 2nd values) showed a significantly better MFS than patients with value changes or continued high NLR and PLR. In particular, patients with continued high 2nd NLR or PLR cutoff values had significantly poorer MFS than patients with continued low NLR or PLR cutoff values (Fig. 2c and d).

## Discussion

This study demonstrated that patients with persistent high NLR and PLR after initial treatment have significantly worse prognosis with regard to late metastasis. In particular, we demonstrated that the NLR and PLR after initial treatment better reflect the prognosis than the NLR and PLR at the time of diagnosis. This result may explain the considerable differences in prognosis in breast cancer patients who have received the same standard treatment.

Tumor development, progression, and metastasis are affected by the host inflammation status and immune response in the tumor microenvironment [13–16]. Numerous studies have shown that lymphocytes play a critical role in tumor immune surveillance [16, 17], and are able to control tumor growth by their cytotoxic activity and induction of apoptosis [18]. Clinical data have shown that an increased density of tumor-infiltrating lymphocytes is associated with favorable prognosis in breast cancer [19, 20]. Meanwhile, neutrophils have been shown to inhibit the immune response by suppressing the cytolytic activity of immune cells, such as lymphocytes, activated T cells, and natural killer cells [21, 22]. Moreover, neutrophils and macrophages have been reported to secrete tumor growth-promoting factors, IL-6, IL-8, including vascular endothelial growth factor, and elastases, and thus likely contribute to a pro-tumor microenvironment [23–26]. Furthermore, platelets have been shown to secrete cellular growth factors, including transforming growth factor beta, platelet-derived growth factor, and vascular endothelial growth factor, which could stimulate tumor proliferation and angiogenesis [27–29]. Therefore, having high NLR and PLR, with a high neutrophil or platelet count and/or low lymphocyte count, can result in poor prognosis of multiple cancers.

A recent meta- analysis examining 100 studies demonstrated that a high NLR is associated with adverse survival in many solid tumors [5]. Similarly, in a meta-analysis of breast cancer, a high NLR was found to be associated with an adverse disease-free survival and overall survival, with a greater association with disease-specific outcome in estrogen receptor and HER-2 negative disease. Furthermore, the PLR in breast cancer highly correlated with clinicopathologic characteristics and was associated with poor prognosis [7].

With well-established prognostic factors, the estimation of risk development of a systemic disease following the treatment for breast cancer can be made possible. Known prognostic factors include histologic subtype of breast cancer, tumor grade, tumor size, involvement of skin or chest wall, extent of involvement of regional lymph nodes, hormone receptor status, and HER-2 status. However, due to the complex nature of breast cancer, the progression and prognosis according to time have been variable and difficult to predict adequately. Recently, a number of proven multigene array expression profiles, such as Oncotype Dx® and Pam-50 ror®, have yielded better predictive power of late recurrence; however, these tests are expensive and inaccessible to most patients [30–32].

In recent years, considerable effort and resources have been used in developing biomarkers, which can help to individual tailor therapy for cancer patients. A small number of patients have persistent poor clinical outcome irrespective of treatment with standard therapy; thus, finding a marker that predicts the prognosis of these patients remains a valuable research subject. Changes in blood inflammatory markers might be useful to predict the post-treatment prognosis and tailor the therapy after. Previous small studies have shown that chemotherapy can normalize an elevated NLR early after the initiation of treatment and that patients with a normalized NLR may have improved clinical outcome in advanced colorectal, urothelial, and biliary cancer [33–35]. Thus, it is considered that the prognostic role of the NLR might still be relevant for the evaluation of the early effects of systemic therapy. Further, in patients with metastatic breast cancer, high NLR was found to be factor related to low responsiveness to eriburin-based treatment [36]. Recently inflammatory markers were also reported as important prognostic markers not only in systemic therapy but also in immune therapy. In study, which perfromed in 90 patients who received immunotherapy based treatment regimens, elevated baseline and early increases in NLR and PLR values were strongly associated with poor clinical outcomes in advanced cancer patients [37].

In most cancer patients, a routine blood test is widely used as a traditional examination test at the time of diagnosis and follow-up periods. The results of our study confirmed that observing the process of continuous change, as well as the initial NLR or PLR, can also be an important indicator for predicting the prognosis of the patient. Indeed, a recent study demonstrated that patients with a high NLR approximately 5 years after the initial diagnosis had significantly worse breast cancer-free survival with late recurrence (HR, 1.448; *p* < 0.001). Furthermore, it was shown that the NLR obtained after the completion of primary treatment can predict later recurrence in breast cancer patients [38].

Our study has several limitations. First, the retrospective protocol of this study necessitates prospective validation of the prognostic effect. Second, this study analyzed the NLR and PLR values between 2 and 3 years, but further research is needed to determine whether the prognosis varies after this period, depending on the pattern of continuous change in long term. Third, we only analyzed our hospital data, which included a relatively small number of enrolled patients and had an insufficient follow-up period.

## Conclusions

We showed that NLR and PLR follow-up values are important predictors of prognosis in breast cancer patients. NLR and PLR changes are easily accessible markers with a simple blood test follow-up, so they should be considered as potential prognostic biomarkers to be associated with others.

## Data Availability

All the data supporting our findings are contained within the manuscript.

## Abbreviations

NLR: Neutrophil-to-lymphocyte ratio
PLR: Platelet-to-lymphocyte ratio
ROC: Receiver operating characteristic
MFS: Metastasis-free survival
AUC: Area under the ROC curve
